# Red-capped mangabeys (*Cercocebus torquatus*) adapt their interspecific gestural communication to the recipient’s behaviour

**DOI:** 10.1038/s41598-020-69847-6

**Published:** 2020-07-30

**Authors:** Juliette Aychet, Pablo Pezzino, Arnaud Rossard, Philippe Bec, Catherine Blois-Heulin, Alban Lemasson

**Affiliations:** Univ Rennes, Normandie Univ, CNRS, EthoS (Éthologie animale et humaine) - UMR 6552, 35000 Rennes, France

**Keywords:** Social behaviour, Animal behaviour

## Abstract

Sensitivity to recipient’s attention and responsiveness are critical markers of intentional communication. Although previous research showed that ape gestures can be intentional, few studies have yet addressed this question concerning monkeys. Here, we characterise the effect of a recipient’s presence, attentional state and responsiveness on the interspecific gestural communication of captive red-capped mangabeys (*Cercocebus torquatus*). Previous reports showed that they produced learnt begging gestures towards a human recipient preferentially when the latter was facing them. We used here a novel setup that allows subjects to move around an experimenter and to use different modalities (visual and acoustic) to communicate. We found that when the recipient was not facing them, mangabeys moved to a position in the visual field of their recipient rather than using attention-getters. Interestingly, unlike apes, they did not elaborate their communication visually or acoustically when the experimenter did not respond favourably to their begging. However, our results may suggest that begging gestures were goal-directed, since mangabeys inhibited them when the experimenter was not available to answer immediately (i.e. give a reward). Overall, red-capped mangabeys’ interspecific visual communication presented intentionality features, but their use of begging gestures was less flexible than that of great apes in similar situations.

## Introduction

Intentionality of non-human primate communication is a key feature for the study of language origins^1,2^. The first degree of intentionality, first formulated by Dennett^3^ and studied in animal communication, corresponds to a signal voluntarily transmitted by a signaller toward a recipient in order to change the latter’s behaviour^4,5^, the signaller choosing between different strategies to achieve its social goal^6^. Behavioural indicators, mainly adapted from human developmental studies (e.g.^7^), enable the assessment of the intentionality of a communication signal, notably by evaluating whether the signaller acts as if the signal is social- and goal-directed or not^8–10^. Among these indicators, the social use of the signal, the sensitivity to and manipulation of recipient’s attentional state, as well as the goal-dependent persistence and elaboration in signalling, are critical markers of intentional communication.

Therefore, intentionality may be reflected by the effect that the presence and characteristics of a recipient may have on the communication, including the adaptation of the signaller to the recipient’s attentional state or its understanding of the signal (e.g.^8–11^). Studies addressing this topic on primate gestural communication have focussed mainly on apes, showing that their gestures can be intentional (see reviews:^5,12,13^), while few studies focus on other non-human primate species^9,14^. Intentionality markers accompanying non-human primates’ gestural communication can be studied by observing intraspecific interactions (e.g.^15–20^), however control of the recipient’s behaviour cannot be applied practically in this context to evaluate rigorously the effects of different characteristics. Consequently, intentionality of non-human primate gestures is often investigated at an interspecific level through an experimental approach, using setups in which subjects produce learnt begging or pointing gestures directed towards a human, in order to obtain a food reward (e.g.^21–33^).

The social use of begging gestures, that is, the effect of the mere presence of a recipient on the production of the gestures, is a requirement to qualify them as communicative, and thus to study their intentionality^9,10,12,34,35^. It has been evidenced in great apes (chimpanzees, *Pan troglodytes*, bonobos, *Pan paniscus*, orangutans, *Pongo pygmaeus*, gorillas, *Gorilla gorilla*: e.g.^21,24,34,36^), but also in several monkey species (Tonkean, rhesus and bonnet macaques, *Macaca tonkeana*, *M. mulatta* and *M. radiata*^31,33,37,38^; olive baboons, *Papio anubis*^28,29^; capuchins, *Cebus apella*^39,40^; and squirrel monkeys, *Saimiri sciureus*^26^). Moreover, pointing and begging gestures in great apes are accompanied by gaze alternations between the recipient and the food^21,35,36,41,42^, considered an indicator of intentional communication acquisition during human development^43,44^. This has been evidenced also in some monkey species’ interspecific gestures (i.e. squirrel monkeys^45^; olive baboons^46,47^; Tonkean and rhesus macaques^31,38^).

While the effect of recipient’s presence reveals the social-dependency of a signal, sensitivity to recipient’s attentional state also reflects the fact that signaller intends to direct its signal in order to reach its goal^9,10^. In that regard, Roberts et al.^48–50^ suggested that intentional gestures themselves evolved from the need to direct effectively the recipient’s attention to the goal. To adapt its gestural communication to its recipient’s attentional state, a signaller can use different modalities (visual, acoustic, tactile) or display attention-getters before producing a visual gesture^51^. The first demonstration was made by Tomasello and colleagues^52^, based on observations of chimpanzees displaying different gestural modalities depending on the recipient’s visual attentional state. Then this trait was reported for all great ape genera based on both observational and experimental data (e.g. chimpanzees^19,22,34,53,54^; bonobos^15,55^; orangutans^16,20,56^; and gorillas^18,56^). Another strategy to deal with recipient’s attentional state is to move to a position in the recipient’s visual field before gesturing. Reports showed that chimpanzees moved so that they could stay in front of their human recipient before gesturing^23^, and a recent study demonstrated this ability in wild bonnet macaques^33^. To our knowledge, this has not yet been investigated in other catarrhine or platyrrhine monkeys, although several species are known to be sensitive to a recipient’s attentional state for displaying begging gestures, based on the human’s head or body posture (olive baboons^28^; red-capped mangabeys^27^; Tonkean and rhesus macaques^31,38^; capuchin monkeys^39,40^).

Moreover, the flexibility of monkeys’ gestural communication regarding their strategy to deal with their recipient’s response has been poorly studied (but see recent reports on olive baboons^57^ and bonnet macaques^58^). Indeed, after a signal has been emitted the recipient can either respond favourably regarding the signaller’s goal, not respond at all, or respond in an incongruent way. In an intentional communication event, a signaller can be expected to act strategically in order to reach its goal, and so to persist (by repeating the signal) or to elaborate its communication (by modulating the signal or using new ones) when the recipient does not give a satisfying answer^9,10^. Both persistence and elaboration were brought to light in great apes^12,13,59,60^, notably using experimental paradigms of interspecific communication where a human either does not give a response or gives a partial response to an ape’s begging gestures^8,25^.

In the current study, we addressed all these aspects of intentionality on the gestural communication of a still poorly studied species of catarrhine monkeys, the red-capped mangabey (*Cercocebus torquatus*), via an experimental approach in captivity. An observational study previously showed that red-capped mangabeys’ gestures were adapted to their recipient’s attentional state and accompanied by other indices of intentionality such as goal persistence [Schel et al., under review]. Moreover, a first study in controlled conditions showed that red-capped mangabeys produced learnt begging gestures towards a human experimenter preferentially when the latter was facing them^27^. Our aim here is to characterise more precisely the gestural communication strategies that mangabeys adopt to deal with changes of the recipient’s attentional state, and complete the previous findings by evaluating other aspects of intentional communication. For this purpose, we used a novel experimental setup that allowed the subjects to move freely around the experimenter and to use different sensory modalities (visual and audible) to communicate. First, we investigated the social-dependency of learnt begging gestures in red-capped mangabeys, in order to confirm that they were communicative. We hypothesised that (i) red-capped mangabeys would produce them only in the presence of a human recipient and (ii) would display gaze alternation while gesturing. Second, we studied the effect of changes in the recipient’s attentional state, based on change of head and body postures. We hypothesised that mangabeys (iii) would produce less begging gestures in front of an inattentive than an attentive recipient, (iv) would position themselves in the visual field of their recipient to communicate, or (v) would use audible attention-getters when gesturing in front of the recipient was not possible. Third, we tested the effect of different recipient’s response on the gestural communication of mangabeys, hypothesising that (vi) they would persist in gesturing if the recipient did not respond favourably to their begging, or (vii) would elaborate their communication visually (by changing their gesture amplitude, or producing other visual signals) or acoustically (by producing audible gestures or vocalisations).

## Methods

### Ethical note

The current study and its experimental procedure have been approved by the Rennes Ethical Committee for Animal Experiment (Authorization N°2019012915271260). The living conditions of the captive mangabeys at the Station Biologique de Paimpont (University Rennes 1, France) follow all applicable international, national and institutional guidelines for the care and use of animals, and are regularly monitored by the local responsible authorities [Housing agreement for research D35-211-18, delivered by the “Direction Départementale de la Cohésion Sociale et de la Protection des Populations” (DDCSPP)].

### Subjects and housing conditions

We tested the effect of a recipient’s presence and characteristics on the gestural communication of fifteen red-capped mangabeys (*C. torquatus*). Our subjects were ten females and five males between 4 and 31 years old (Table 1), living in social groups of two to thirteen individuals, whose compositions had been stable for at least 20 months. These mangabeys were housed in outdoor-indoor enclosures of different sizes (from 8 to 26.4 m^2^ for indoor enclosures, 14.7–37.2 m^2^ for outdoor enclosures, and height from 2.5 to 4.4 m). Indoor temperature was maintained at 22 °C, and enclosures were enriched with wood and metal perches, chains and hessian ribbons. The floor of the indoor enclosures was covered with straw and sawdust, while the floor of the outdoor enclosures was covered with cement or bark. Mangabeys were fed twice a day with fresh fruits, vegetables and monkey chow, and water was available ad libitum.

**Table 1:** Characteristics of our red-capped mangabey subjects.

Social group	Subject	Sex	Date of birth	Test location	Previous training experience:	
					Pointing gestures^72,80^	Begging gestures^27^
I	Bell	Female	31/03/2002	A	Trained	Naive
	Chipie	Female	28/06/1992	A	Trained	Trained
	Chipse	Female	03/01/2006	A	Trained	Trained
	Gofrette	Female	08/11/1996	A	Trained	Trained
	Joly	Female	22/10/2000	A	Trained	Naive
	Julie	Female	08/05/2004	A	Trained	Trained
	Kargi	Male	19/05/2005	A	Naive	Naive
	Litchi	Male	20/04/2015	A	Naive	Naive
	Maillette	Female	29/12/2009	A	Trained	Naive
	Many	Female	14/08/2008	A	Trained	Naive
	Triskelle	Female	21/04/2015	A	Naive	Naive
	Zunie	Female	03/07/1987	A	Trained	Trained
II	Roby	Male	18/11/2010	B	Naive	Naive
III	Coët	Male	31/08/2011	B	Naive	Naive
	Tips	Male	10/07/2011	B	Naive	Naive

These experiments were conducted between January and April 2019, in the morning or in the afternoon, always before feeding. The tests took place in two outdoor enclosures (A and B, Table 1), depending on the focal social group, each time in the subjects’ home cage or an adjacent one. Subjects were trained and tested alone, in physical isolation from their social group but this setup allowed visual and auditory contact with their conspecifics.

### Experimental setup

**Figure 1:**
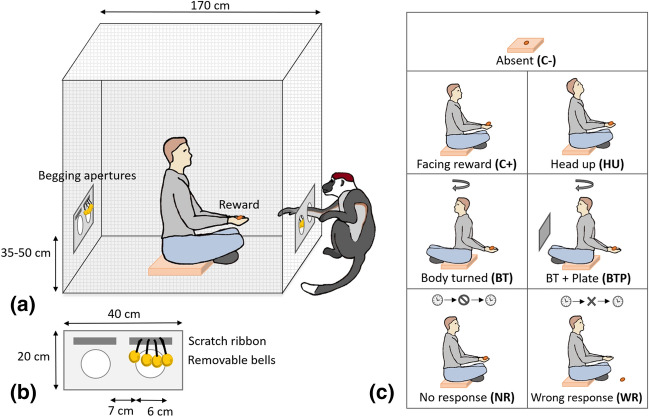
Experimental setup and procedure. (**a**) The experimenter was placed in a cubic cage in the centre of the test area and presented a peanut in his hands. The mangabey could thus beg for the reward and move freely around the experimenter. (**b**) Begging-plates were fixed on two opposite sides of the cage, with two apertures above which it was possible to fix a row of bells so that begging gestures would be audible. (**c**) The presence and attentional state of the experimenter, as well as his responsiveness (i.e. rewarding behaviour) varied with the experimental condition.

The experimenter was placed in a cubic cage (1.70 × 1.70 × 1.70 m), built for the occasion, in the centre of the test area, thus allowing the subjects to move freely around the experimental setup (Fig. 1a). The experimenter was seated on a 20 cm-high platform, and was able to turn his body towards one side of the cage or another. The metallic grid composing the cage was tight enough to prevent the mangabeys from sticking an arm directly through it (dimensions 2 × 2 cm). Plates of Plexiglas (20 × 40 cm) were hooked on two opposite sides of the cage, with two apertures (diameter 6 cm, 7 cm apart horizontally) through which mangabeys could produce begging gestures (Fig. 1b). The height of the begging-plates was adjustable in relation to the size of the subject (i.e. positioned at the level of the subject’s shoulders: 35 cm for juveniles and adult females, and 50 cm for adult males). Velcro strips were placed above ‘begging apertures’, allowing the experimenter to fix a row of four hanging metallic bells, thus begging through the corresponding aperture was audible. During the tests, the bells were hung only above one aperture of each begging plate, so that the mangabeys could chose to beg through an aperture with or without bells. Moreover, an opaque PVC plate could be fixed on the apertures, to prevent begging gestures on one side of the cage.

### Training and habituation

#### Begging gesture training

Begging gestures studied here correspond to an extension of the arm with hand open in the direction of a recipient in order to obtain a reward. Given that red-capped mangabeys do not naturally produce this type of gesture, all our subjects were trained to produce them before conducting the tests. Some subjects had already been trained to produce pointing and/or begging gestures for previous experiments and others were naive (Table 1), yet we used the same methodology for all individuals, ritualizing reaching actions into gestures. Applying the same procedure as in previous studies^28,29,31^, each training session was composed of three steps during which the experimenter presented a peanut in front of the mangabey, increasing progressively the distance between the reward and the subject. First, the mangabey was able to grasp the reward directly on the hand of the experimenter; second, the reward was held at the limit of the subject’s reach, and given as soon as the extension of the arm was produced; finally, the peanut was out of subject’s reach and was given only when the arm was extended towards it with hand open, without any grasping movement. Daily training sessions of 5–20 min were performed until the criterion of two consecutive sessions with only valid begging gestures was reached (between 2 and 5 sessions were needed, depending on the subject), and were then regularly performed until the experiments took place. Begging gestures were considered valid if they did not imply any rotation of the subject’s body to reach the reward and any grasping actions at the end of arm extension. Training sessions were performed in the indoor enclosure of the subjects’ home cage, using the same begging-plates as the ones used subsequently during the experiments, but without the bells (Fig. 1b).

#### Habituation to the bells

Once the mangabeys had acquired the begging gesture, we habituated them to beg with bells hanging in front of the begging apertures, for them to experiment the sound production resulting from the manipulation of the bells, and to reduce neophobia of these objects. Thus, rows of bells were hung above both apertures of the begging-plates (Fig. 1b). We followed the same training procedure as before, alternating training sessions with and without bells, and the same criterion of two consecutive valid sessions was required (between 2 and 7 sessions were needed depending on the subject). These training sessions were then repeated regularly until the experiments took place.

#### Habituation to the test area and to the cage setup

Two sessions of 10 min—habituation to the test area and to the cage setup were carried out for each individual. Subjects were introduced individually into the test area where the experimenter was sitting inside the cage, presenting a peanut in his hand in front of one of the begging-plates. Subjects could explore the area freely and were encouraged to produce begging gestures, as during the trainings, alternatively through the two accessible sides of the cage.

### Experimental procedure

The experimenter sat in the centre of the cage and presented a peanut in his hands at an unreachable distance from the ‘begging apertures’ (50 cm), hands always joint and approached at the same time to the subject when rewarding him, to avoid any laterality bias. The experimenter’s head and body positions and his rewarding behaviour varied with the experimental condition (Fig. 1c). Each subject was exposed to seven different experimental conditions. At the end of each trial, the subject was rewarded regardless of its response. All test trials started when the mangabey positioned itself in front of one begging plate, where it had been rewarded during the preceding trial to standardize the beginning of the trials.

During the positive control condition (**C+**), the experimenter held a reward in front of him in a standardized position (as during training), with his head and eyes directed towards the subject. The reward was given to the mangabey after 10 s, regardless of its gestural communication^27^. The “head up” condition (**HU**) was the same as the positive control condition and lasted 10 s, but the experimenter had modified his attentional state by changing the direction of his head and eyes, to look upwards. In the “body turned” (**BT**) condition, the experimenter also modified his attentional state by turning his body and head in the opposite direction, but maintained the reward on the same side, i.e. holding the peanut behind his back. After 10 s, the experimenter moved back to his initial position and rewarded the subject. To evaluate mangabeys’ use of attention-getters when they were not able to communicate facing their recipient, we presented a condition where the experimenter’s body and head were turned away from the subject and the ‘begging apertures’ in front of the experimenter were blocked with an opaque plate (**BTP** condition). The “no response” condition (**NR**) was similar to the positive control, except that the experimenter did not respond (i.e. give the reward) after the first 10 s (NR.a), and waited instead for 10 supplementary seconds (NR.b) before rewarding the subject. During the “wrong response” condition (**WR**), the experimenter mimicked giving a reward after 10 s (WR.a) and then took up again the same position as in the positive control condition for 10 more seconds (WR.b) to evaluate whether the subjects would persist or elaborate their begging gestures after not receiving the expected response. In this condition, in order to carry the exact same action of rewarding but without making the peanut reachable to the monkey, the reward was put on the ground in front of the experimenter instead of in the experimenter’s hands (see Supplementary Video S1 for examples of each experimental condition).

Each mangabey was subjected to two experimental sessions (2 × 9 trials in a row), each one included the six abovementioned conditions plus three “motivational trials” (as in Maille et al.’s study^27^) presented in a random order. Motivational trials were similar to the positive control trials but, contrarily to the experimental trials, the subjects were rewarded as soon as they produced a begging gesture. Moreover, before or after each session, we carried out a negative control trial (**C**−) for which the experimenter was absent but a reward was visible in the centre of the cage, to evaluate whether begging gestures would be performed in the absence of a human recipient. The two experimental sessions were separated by 1–12 days according to the subject.

We randomized the order of the conditions within a session, and semi-randomized the order in which the individuals were tested (taking into account subjects’ social groups and dominance rank within groups). Moreover, we balanced, between sessions of each subject, the position of the bells (above the left or right begging aperture), the direction of rotation of the experimenter when he turned his body (towards the left or towards the right), and the order of the negative control trial (before or after presentation of the other conditions).

### Data collection

#### Video recording and coding

The experimental sessions were recorded from inside the test cage by two GoPro White Hero 7 cameras attached to the experimenter’s chest and back, so that monkeys were visible on videos of both ‘begging sides’ of the cage. Moreover, a JVC Full HD GZ-RX615 camcorder was positioned outside the test area. Videos were then extracted and coded by a first observer using the software BORIS v.5.1.0^61^. Videos taken from inside the cage were used to code the gestures and gazes of the mangabeys during the experiment, and the videos taken from outside were used to code the movements of the subjects around the cage. The detailed variables collected are presented in Table 2. In order to characterize mangabeys’ begging gestures depending on experimental conditions, as well as the potential communication elaboration or use of attention-getters, we recorded: the number of begging gestures produced, the latency for first gesture, the gesture amplitude, the aperture chosen to beg and the number of any other communicative signals. We quantified gaze alternations, which are indicator of intentional communication^43,44^, and upward gazes, which were considered as indices of monkeys’ perception of the experimenter’s attentional state in HU condition. Finally, in order to assess whether mangabeys preferred to position themselves in the visual field of their recipient, we quantified the time spent in different locations, and recorded whether they circumvented the cage and on which side.

**Table 2:** Behavioural variables studied.

Behaviour category	Variable	Variants	Description
Begging gestures	Number of begging gestures	–	Extension of arm through one begging aperture, with hand open and without grasping movement
	Latency for first begging gesture (s)	–	Duration between the start of the experimental trial and the initiation of the first begging gesture
	Begging gesture amplitude	Amplified (Beg+)	Begging gesture amplified so that the entire arm passed through the begging aperture and the arm moved up in front of the experimenter, still without grasping movement of the hand
		Normal	Begging gesture mangabeys had been trained to produce, with forearm extended through the begging aperture, without grasping movement of the hand
		Lessened (Beg−)	Begging gesture with arm not very extended, so that only the hand passed through the begging aperture
	Begging aperture chosen	With/without bells	Begging aperture through which the arm is extended when producing the begging gesture
Other signals	Number of other communication signals	–	Vocalisations^81^, gestures (Schel et al. *under review*) or facial expressions (Aychet et al. *under review*) directed towards the experimenter
Gazes	Number of gaze alternations	–	Three consecutive gazes in a row (i.e. two eye movements) directed alternatively towards the experimenter’s face and the reward
	Number of gazes upwards	–	Head and eyes directed upwards
Displacements	Time spent in different locations (s)	Front	Time the subject spent in front of the experimenter
		Back	Time the subject spent behind the experimenter
		Away	Time spent away from the begging sides of the cage, i.e*.* > ± 45° from the front of the begging sides
	Change of begging side	Yes/no	Movement around the cage to switch begging side during a trial
	Side chosen to circumvent the cage	Same/different	Side chosen to circumvent the cage being the same or not as the side the experimenter chose to turn his body in BT and BTP conditions

#### Inter-observer reliability

In order to assess the reliability of the video coding, a second observer coded the behavioural variables for eight random experimental sessions, corresponding to 27% of the total amount of data. We computed Kendall’s coefficients of concordance for the quantitative variables (gazes, begging gestures, latency for first gesture, time spent in different locations) and Cohen’s kappa coefficients for the qualitative one (gesture amplitude)^62^. A strong agreement^63,64^ was found between the two observers (Gaze alternations: *W* = 0.833, *P* < 0.001; Upward gazes: *W* = 0.886, *P* < 0.001; Begging gestures: *W* = 0.958, *P* < 0.001; Latency for first gesture: *W* = 0.882, *P* < 0.001; Time spent in front, in the back of the experimenter and away from begging sides: *W* = 0.944, *P* < 0.001, *W* = 0.964, *P* < 0.001 and *W* = 0.919, *P* < 0.001 respectively; Gesture amplitude: κ = 0.841, , *P* < 0.001).

### Data analysis

We used R. 3.5.0 software^65^ to run all statistical tests and models. All tests were two-tailed and alpha-level was always set at 0.05. First, to assess the effect of the recipient’s presence and attentional state, we compared the mangabeys’ behaviour between C− (absence), C+ (presence), HU (head up), BT and BTP (body turned without and with opaque plate) conditions. Then, we analysed the effect of the recipient’s responsiveness. Within the NR condition, we compared the first 10 s (NR.a) and the last 10 s (NR.b) to assess the effect of receiving no response from the recipient on the mangabeys’ communication gestures. Similarly, C+, WR.a and WR.b conditions were compared to assess the effect of a wrong response from the recipient.

Therefore, generalized linear mixed models (GLMM) and linear mixed models (LMM) were used to analyse the following behavioural variables depending on the relevant experimental conditions (see Supplementary Table S1 for detailed list of models used): number of begging gestures, latencies for first begging gestures, proportions of inhibited and amplified begging gestures, proportions of audible begging gestures, change of begging apertures when gesturing, number of gaze alternations, number of upward gazes, as well as the time that mangabeys spent in front or behind the experimenter, or away from the begging sides of the cage. In each statistical model, the individual, the experimental session and the order of the different conditions within the session were taken into account as random effects. Model quality was assessed by verifying the normality of the residuals, using normal probability plots. When comparing more than two conditions, p-values were adjusted for multiple comparisons using the “False Discovery Rate” (FDR) method, controlling the proportion of false significant p-values^66^.

We tested the correlation between the number of begging gestures and the number of gaze alternations using a Spearman correlation test. Finally, the proportions of individuals who circumvented the cage when the experimenter turned his body, and the proportions of individuals who followed the same side as the experimenter when circumventing the cage, were all tested to differ from a random distribution using exact binomial tests, and were compared between conditions using Fisher’s exact tests for count data.

## Results

### Effect of presence and attentional state of the recipient

#### Presence of a recipient

**Figure 2:**
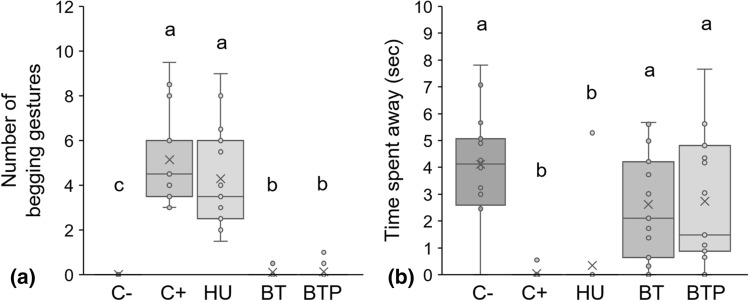
(**a**) Number of begging gestures produced and (**b**) time spent away from the ‘begging side’ of the cage (in seconds) in relation to the experimenter’s presence and attentional state. Individual data are plotted as means of sessions A and B. *C−* experimenter absent, *C+* experimenter facing reward and subject, *HU* head up, *BT* body turned, *BTP* body turned and opaque plate blocking begging apertures in front of the experimenter. GLMM negative binomial was used to analyse the number of begging gestures (**a**) and LMM to analyse the time spent away from the ‘begging side’ of the cage (**b**). Different lower-case letters indicate significant differences between conditions (*P* < 0.05).

**Figure 3:**
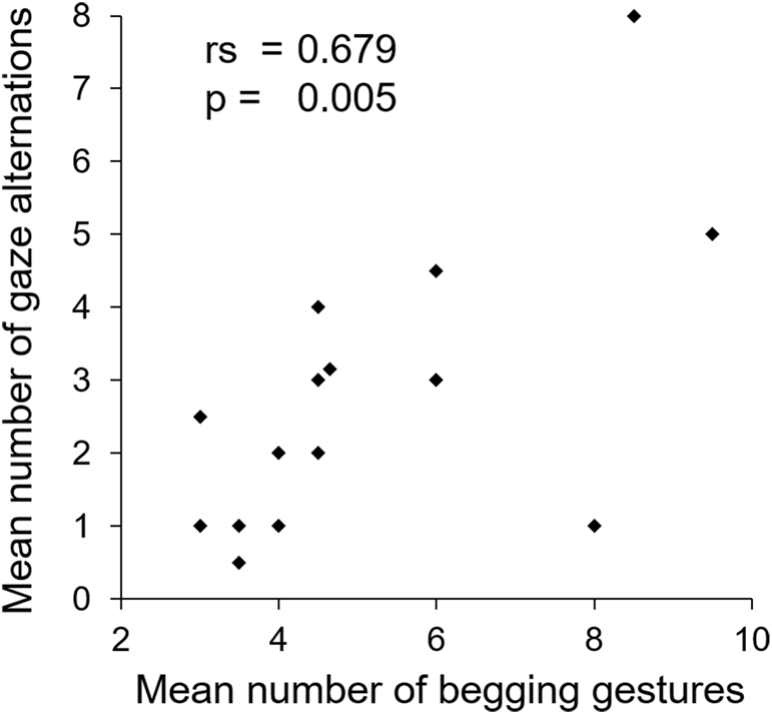
Begging gestures produced by mangabeys in relation to number of gaze alternations between the experimenter and the reward in the positive control condition.

Red-capped mangabeys produced approximately 4.5 begging gestures (Median ± *IQR* = 4.5 ± 2.5) per trial when the experimenter was present and facing them (C+), while no begging gestures were produced when the experimenter was absent (Fig. 2a, GLMM negative binomial C− vs C+: *Z* = − 3.915, *P* < 0.001. See Supplementary Table S2 for detailed results of all models). Moreover, when the experimenter was absent, the subjects spent more time away from the ‘begging side’ of the cage than during the C+ condition (Fig. 2b, LMM C− vs C+ : *t* = 7.095, *P* < 0.001). Furthermore, the number of begging gestures produced during the C+ condition was significantly positively correlated with the number of gaze alternations between the reward and the experimenter (Fig. 3, Spearman correlation: *S* = 179.65, *rs* = 0.679, *P* = 0.005).

#### Attentional state of the recipient: change of head position

**Figure 4:**
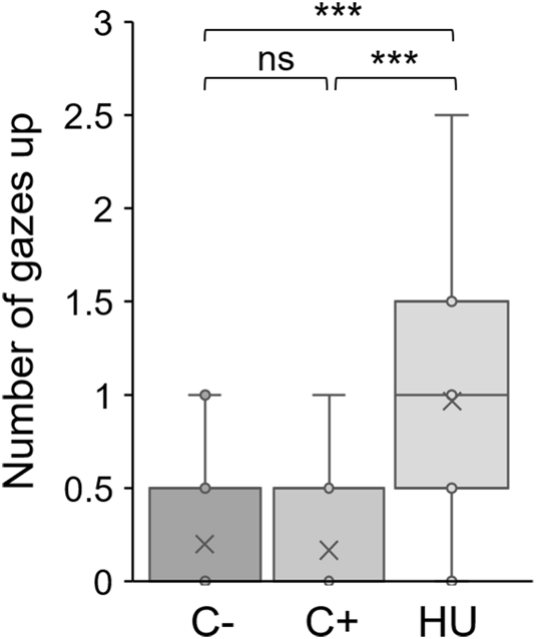
Upward gazes in relation to the experimenter’s presence and head position. Individual data are plotted as means of sessions A and B. *C*− experimenter absent, *C+* experimenter facing reward and subject; HU: Head up. GLMM Poisson: ***P < 0.001; *ns* non-significant difference.

Neither numbers of begging gestures (GLMM negative binomial C+ vs HU: *Z* = 1.487, *P* = 0.196) nor latencies before first begging gesture differed significantly between C+ and HU (Fig. 2a, GLMM Gamma C+ vs HU: *t* = − 0.247, *P* = 0.960). However, mangabeys exhibited significantly less gaze alternations when the experimenter held his head up than in the control condition (GLMM Poisson C+ vs HU: *Z* = − 2.679, *P* = 0.007), and they exhibited more upward gazes (Fig. 4, GLMM Poisson C+ vs HU: *Z* = 3.630, *P* < 0.001).

#### Attentional state of the recipient: change of body position

Red-capped mangabeys produced few or no begging gestures when the experimenter turned his body away (Fig. 2a, GLMM negative binomial BT vs C+: *Z* = − 6.954, *P* < 0.001, BT vs C−: *Z* = − 2.865, *P* = 0.006), and there were no significant differences between BT and BTP conditions (GLMM negative binomial BT vs BTP: *Z* = 0.417, *P* = 0.846). Almost all individuals circumvented the cage when the experimenter turned his body, despite the position of the reward (Exact binomial tests: *P* < 0.001 for BT and BTP), while none of them changed side during C+ trials (Fisher exact tests BT vs C+: *P* < 0.001, BT vs BTP: *P* = 1.000). Thus, red-capped mangabeys spent more time away from the ‘begging sides’ of the cage during BT and BTP than during C+ trials (Fig. 2b, LMM BT and BTP vs C+: *t* = 5.231 and *t* = 5.434, *P* < 0.001), but it tended to be less than during C− trials (LMM BT and BTP vs C−: *t* = 2.554, *P* = 0.062, and *t* = 2.409, *P* = 0.066). Moreover, when circumventing the cage, individuals preferred to use the same side as the experimenter (Exact binomial tests: *P* = 0.035 for BT and *P* = 0.007 for BTP). Taking into account all conditions, subjects spent more time in front of the experimenter than behind him (LMM ‘Front’ vs ‘Back’: *t* = 13.607, *P* < 0.001).

### Effect of recipient’s responsiveness

#### No response

**Figure 5:**
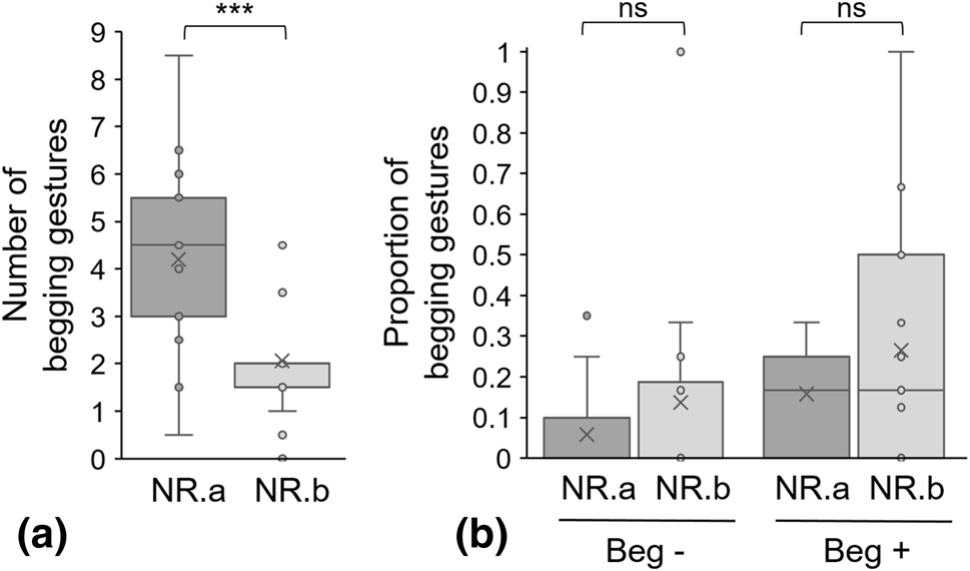
(**a**) Begging gestures and (**b**) proportions of lesser (Beg−) and amplified (Beg+) begging gestures when the experimenter did not answer. Individual data are plotted as means of sessions A and B. *NR.a*: first 10 s; *NR.b*: last 10 s. GLMM Poisson was used to analyse the numbers of begging gestures and LMM to analyse the proportions of lesser and amplified begging gestures. ***P < 0.001; *ns* non-significant difference.

After 10 s without a response from the experimenter, subjects produced less begging gestures (Fig. 5a, GLMM Poisson NR.a vs NR.b: *Z* = − 4.624, *P* < 0.001), and the number of gaze alternations decreased (GLMM negative binomial NR.a vs NR.b: *Z* = − 2.502, *P* = 0.012). The amplitude of the gestures did not vary when the experimenter did not answer (Fig. 5b, LMM NR.a vs NR.b: *Z* = 1.082, *P* = 0.285 for Beg−, *Z* = 1.619, *P* = 0.114 for Beg+). Moreover, when comparing NR.a and NR.b, subjects did not use more the ‘begging apertures’ with bells (LMM NR.a vs NR.b: *t* = 0.153, *P* = 0.879) and did not produce other communication signals in the direction of the experimenter (visual or vocal).

#### Wrong response

**Figure 6:**
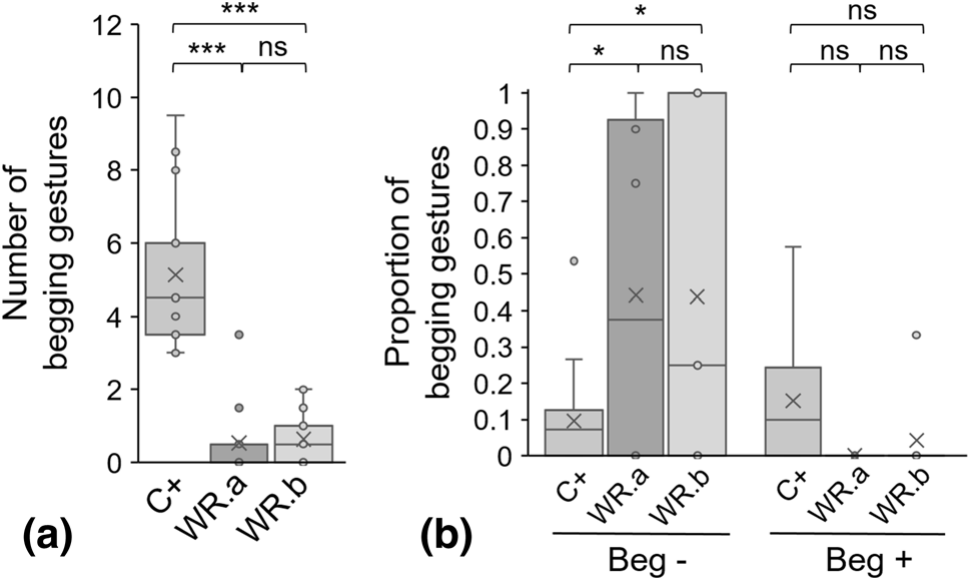
(**a**) Number of begging gestures and (**b**) proportion of lesser (Beg−) and amplified (Beg+) begging gestures when the experimenter gave a wrong response. Individual data are plotted as means of sessions A and B. *WR.a*: first 10 s; *WR.b*: last 10 s, after a false response. GLMM Poisson was used to analyse the number of begging gestures (**a**), and LMM to analyse the proportion of lesser and amplified begging gestures (**b**). ***P < 0.001; *P < 0.050; *ns* non-significant difference.

Numbers of begging gestures produced by the subjects when the experimenter gave a wrong response, i.e. mimicked rewarding them (Fig. 6a, GLMM Poisson WR.a vs WR.b: *Z* = 0.511, *P* = 0.610), and latencies before first begging gesture (GLMM Gamma WR.a vs WR.b: *t* = − 0.016, *P* = 0.988) did not differ significantly between WR.a and WR.b. In addition, amplitude of gestures did not vary significantly between WR.a and WR.b (LMM WR.a vs WR.b: *t* = − 0.595, *P* = 0.556 for Beg−, *t* = 0.616, *P* = 0.543 for Beg+) and none of the subjects used the begging aperture with bells or produced other communication signals directed towards the experimenter.

In fact, very few begging gestures were produced in these situations, i.e. when the reward was on the ground in front of the experimenter instead of in his hands (Fig. 6a, GLMM Poisson WR.a vs C+: *Z* = − 8.577, *P* < 0.001), and latencies before first beg thus increased (GLMM Gamma WR.a vs C+: *t* = 5.357, *P* < 0.001). Moreover, the gestures produced then were of lesser amplitude than during positive control trials (Fig. 6b, LMM WR.a vs C+: *t* = 2.978, *P* = 0.014), and we observed less gaze alternations (GLMM Poisson WR.a vs C+: Z = − 5.220, *P* < 0.001).

## Discussion

The present study aimed to characterise the effect of recipient’s presence, attentional state and responsiveness on red-capped mangabeys’ interspecific gestural communication. We showed that the production of learnt begging gestures depended on the presence and attentional state of the recipient, and our results helped refine our knowledge on the visual cues used by mangabeys to perceive their recipient’s attentional state. We found that when the recipient was not facing them, mangabeys moved to a position in their recipient’s visual field. Interestingly, unlike great apes, our mangabeys did not elaborate their communication visually nor acoustically when the experimenter did not respond or responded incongruently to their begging gestures. Finally, our results suggest that, when the recipient was not immediately available to answer (i.e. to give a reward), red-capped mangabeys inhibited their begging gestures, which may indicate the goal-directedness of these displays.

The fact that begging gestures were produced only in the presence of the experimenter demonstrates that they are for communication^34,35^. Moreover, the fact that they were accompanied by gaze alternations between the recipient and the referent (i.e. reward) may reflect red-capped mangabeys’ ability to master joint attention, which is notably associated with intentional communication in human infants^43,67^. In this way, mangabeys may indeed direct the attention of the experimenter toward the food reward^50^. This corroborates previous results showing that gaze alternations accompany learnt begging or pointing gestures in other non-human primate species (e.g., orangutans^21^; chimpanzees^35,36^; olive baboons^28,29,47^; rhesus and Tonkean macaques^31,36^; squirrel monkeys^45^). In olive baboons, the frequency of gaze alternations during requesting-gesture events was not associated with an individual’s communication experience with humans^46^. Thus, we assume that present results on gaze alternation in red-capped mangabeys were not conditioned by training but reflect features of spontaneous visual communication.

Red-capped mangabeys produced begging gestures regardless of the upward direction of the recipient’s head and eyes. This was surprising regarding previous results showing that they produced less gestures towards an experimenter when she turned her head laterally^27^. In our experiment, this change of head posture was however discriminated since they produced more upward gazes when experimenter’s eyes and head were directed upwards. Thus, in addition to their ability to follow conspecifics’ gazes^68^, red-capped mangabeys exhibit abilities to follow a gaze in interspecific interactions, as do rhesus macaques^69^. Moreover, we assume that even when subjects perceive the upward head direction of the experimenter, it does not necessarily mean to them that the recipient is unable to see their gestures. In a terrestrial species of monkeys such as red-capped mangabeys, which may keep monitoring their upward environment even during a social interaction, we hypothesise that head posture is not the most significant cue to predict recipient’s incapacity to perceive their visual gesture. Instead, we hypothesise that similarly to gorillas or orangutans, this assessment is mainly based on recipient’s body orientation^56,70^.

Indeed, as Maille and colleagues^27^ showed previously, red-capped mangabeys perceived body posture as a cue of a recipient’s attentional state, as they did not produce any begging gestures when the experimenter turned his back to them. Moreover, this study sheds a new insight into the strategy that mangabeys adopt to deal with this change of recipient’s attentional state. Similarly to great apes^23^ and wild bonnet macaques^33^, red-capped mangabeys moved to position themselves in front of the experimenter, regardless of the position of the reward. They even chose preferentially the same side as the experimenter to turn around the setup, thus avoiding breaking visual contact with him just to be able to attempt to grasp the food. Nevertheless, mangabeys did not produce begging gestures once they had moved in front of the human experimenter in BT condition, and two explanations could account for this. We believe that when the experimenter was facing the subjects but holding the reward in his hands behind his back, the mangabeys did not perceive him as ‘available’ to give the food reward directly, so therefore they did not beg for it. Additionally, together with the fact that mangabeys produced gaze alternations when gesturing, this suggests that begging gestures could be functionally referential to the food reward^35,47,71^, especially considering that previous research highlighted red-capped mangabeys’ ability for referential gestures^72^. One could also say that the production of the learnt begging gesture is just not very flexible and that during BT condition, the experimenter’s posture was too different from the human’s posture during training. Reports on olive baboons suggest that associative learning plays an important role in their ability to discriminate human attention cues, yet all communication features of learnt begging gestures do not rely exclusively on training^73^. Here, mangabeys did gesture in HU condition, in which the experimenter’s posture was also different from the one of training phase, which calls into questions the assumption that associative learning would be the only explanation of our subjects’ behaviour in BT and BTP conditions.

Interestingly, we did not observe any use of vocalisations or auditory gestures as manipulators of the recipient’s attentional state when the latter turned away, even when an opaque plate prevented begging gestures in front of the experimenter. This agrees with observations in macaques (rhesus, Tonkean and bonnet macaques^31,33,38^), gorillas and orangutans^56^, who did not produce vocalisations or auditory gestures as getters of attention in similar interspecific communication contexts. Chimpanzees produce vocalisations preferentially to gestures in front of a visually-inattentive human^22,34^; however in a setup when they had the choice to move into their recipient’s visual field, they did not use any attention-getters^23^. We thus hypothesise that mangabeys did not use visual or auditory attention-getters because they preferred to move directly in front of the human they were interacting with. A supplementary hypothesis is that they do not resort to attention-getters to deal with a recipient’s inattentive state in such contexts, or prefer to use tactile signals instead of auditory signals, as do olive baboons in intraspecific communication^57^, but had no possibility to express them in the present situation.

Mangabeys repeated their gestures in the positive control condition, i.e. when, contrarily to training and motivational trials, they were not immediately rewarded. However, considering the decrease of begging occurrences in NR.b condition, this persistence in gesturing seem to not extend after more than 10 s without receiving a response from the recipient. Moreover, when the experimenter gave an incongruent response, we expected that the subjects would persist in the production of begging gestures, as apes can do^8,25,36,59,74,75^. We noted that red-capped mangabeys produced the same number of gestures before getting any reward (WR.a) and after obtaining a wrong response (WR.b), although few gestures were performed in this specific experimental condition. Since mangabeys did repeat their gestures in the absence of their goal being reached, our hypothesis on communication persistence as a function of recipient’s responsiveness seems partially verified, and two explanations could account to explain that the number of begging gestures decreased with time. This could be due to a decrease in their motivation to communicate with a non-responsive recipient, or be related to the steep hierarchical social system of red-capped mangabeys^76^, in which requesting something for too long from a higher-ranking individual (as could be the experimenter) would be avoided.

Cartmill and Byrne^25^ showed that orangutans do not repeat a gesture that is not efficient, but instead elaborate their communication when they are totally misunderstood by their recipient. Yet, contrary to what we expected based on results for baboons^28^ and apes (chimpanzees^8,23,36,64,74^; bonobos^15^; gorillas^77^ and orangutans^24^), we did not observe any elaboration in the visual or auditory modality when the recipient did not answer. Even if we assume that due to the training and habituation to the bells, red-capped mangabeys were able to understand that bells make a noise and can attract attention, as can do Japanese macaques (*Macaca fuscata*^78^), they did not use them as a way to elaborate their begging gesture through the auditory channel. Overall, one hypothesis could be that red-capped mangabeys’ interspecific communication possesses only a weak flexibility, due to training or to real interspecific differences with great apes. Other explanations of the absence of elaboration could be related to a lack of possibility to elaborate in the tactile modality, or a lack of motivation from mangabeys to elaborate their signalling, since the begging gesture they learnt is already efficient in most cases.

Finally, considering the few begging gestures recorded when the experimenter had his hands behind him (BT and BTP) or when the reward was on the ground instead of held in his hands (WR), we believe that red-capped mangabeys inhibited their begging when the recipient was not directly available to give the reward. Indeed, Kaminski et al.^79^ suggested that the experimenter’s body posture is not only perceived as a cue of attentional state but also encodes disposition to answer. Previous results suggested that great apes^23,56^, bonnet macaques^33^ and capuchin monkeys^39^ produced less begging gestures when the experimenter and the food location were dissociated. Therefore, this inhibition of gesturing might reflect the fact that red-capped mangabeys are less motivated to beg when communication is less likely to be efficient and may suggest the ‘aboutness’ of their begging gestures, in other words their intentionality^3,10^.

## Conclusion

To sum up, this study brings to light the adaptation of red-capped mangabeys’ interspecific visual communication to their recipient’s behaviour. We observed key features of social-directedness in their begging gestures (i.e. gestures depend on the presence and visual attentiveness of a recipient and are accompanied by gaze alternations) and of potential goal-directedness (i.e. gestures depend on the likelihood of recipient to give a satisfying response), which are indices of intentional communication. Furthermore, we refined our knowledge on the visual cues mangabeys perceive as indicators of their recipient’s attentional state, and showed that they prefer moving to maintain visual contact with the recipient instead of using acoustic attention-getters. However, mangabeys did not exhibit any audible or visual elaboration of their interspecific communication when recipient did not respond favourably to their gestures. Future research on this species should investigate to what extent the experimental conditions may affect gesture flexibility, to elucidate the significance of the present results regarding red-capped mangabeys’ ability to gesture intentionally.

## Supplementary information

Supplementary Table 1.

Supplementary Table 2.

Supplementary Table 3.

Supplementary Video 1.

## Data Availability

All data analysed during this study are available as a supplementary table (Supplementary Table S3).
