# Supplementary material for: Red-capped mangabeys (Cercocebus torquatus) adapt their interspecific gestural communication to the recipient’s behaviour
Source: Sci Rep. 2020 Jul 30;10:12843. doi: 10.1038/s41598-020-69847-6 (PMC7393380; doi:10.1038/s41598-020-69847-6)
Supplement: Supplementary file 1 — Supplementary Table 1. [file 41598_2020_69847_MOESM1_ESM.pdf]

Red-capped mangabeys (*Cercocebus torquatus*) adapt their interspecific gestural communication to the recipient's behaviour

Juliette Aychet, Pablo Pezzino, Arnaud Rossard, Philippe Bec, Catherine Blois-Heulin, Alban Lemasson

Supplementary Table S1. Mixed models used to analyse mangabeys' behaviour depending on experimental conditions

C+: Experimenter facing reward and subject; HU: Head up; BT: Body turned; BTP: Body turned and opaque plate blocking begging apertures in front of the experimenter. NR.a: No Response, first 10s; NR.b: No Response, last 10s; WR.a: Wrong Response, first 10s; WR.b: Wrong Response, last 10s; C-: Experimenter absent. GLMM: Generalized Linear Mixed Model; LMM: Linear Mixed Model

Model	Response variable	Random effects	Conditions compared	Model family
		**Effect of recipient's presence and attentional state**		
Model 1	Number of begging gestures	Session, Order of conditions, Individual	C-, C+, HU, BT and BTP	GLMM negative binomial
Model 2	Latency for first begging gesture	Session, Order of conditions, Individual	C+, HU, BT and BTP	GLMM Gamma
Model 3	Number of upward gazes	Session, Order of conditions, Individual	C-, C+, HU	GLMM Poisson
Model 4	Time spent away from begging sides	Session, Order of conditions, Individual	C-, C+, HU, BT and BTP	LMM
		**Effect of no response from the recipient**		
Model 5	Number of begging gestures	Session, Order of conditions, Individual	NR.a, NR.b	GLMM Poisson
Model 6	Proportion of lessened gestures	Session, Order of conditions, Individual	NR.a, NR.b	LMM
Model 7	Proportion of amplified gestures	Session, Order of conditions, Individual	NR.a, NR.b	LMM
Model 8	Proportion of audible begging gestures	Session, Order of conditions, Individual	NR.a, NR.b	LMM
Model 9	Change of begging aperture when gesturing (yes/no)	Session, Order of conditions, Individual	NR.a, NR.b	GLMM Binomial
Model 10	Number of gaze alternations	Session, Order of conditions, Individual	NR.a, NR.b	GLMM negative Binomial
		**Effect of a wrong response from the recipient**		
Model 11	Number of begging gestures	Session, Order of conditions, Individual	C+, WR.a, WR.b	GLMM Poisson
Model 12	Latency for first begging gesture	Session, Order of conditions, Individual	C+, WR.a, WR.b	GLMM Gamma
Model 13	Proportion of lessened gestures	Session, Order of conditions, Individual	C+, WR.a, WR.b	LMM
Model 14	Number of gaze alternations	Session, Order of conditions, Individual	C+, WR.a, WR.b	GLMM Poisson
		**For all experimental conditions**		
Model 15	Time spent in different locations	Session, Individual	In front VS in the back of the experimenter	LMM
