# Supplementary material for: Red-capped mangabeys (Cercocebus torquatus) adapt their interspecific gestural communication to the recipient’s behaviour
Source: Sci Rep. 2020 Jul 30;10:12843. doi: 10.1038/s41598-020-69847-6 (PMC7393380; doi:10.1038/s41598-020-69847-6)
Supplement: Supplementary file 2 — Supplementary Table 2. [file 41598_2020_69847_MOESM2_ESM.pdf]

Red-capped mangabeys (*Cercocebus torquatus*) adapt their interspecific gestural communication to the recipient's behaviour

Juliette Aychet, Pablo Pezzino, Arnaud Rossard, Philippe Bec, Catherine Blois-Heulin, Alban Lemasson

Supplementary Table S2. Results of mixed models

C+: Experimenter facing reward and subject; HU: Head up; BT: Body turned; BTP: Body turned and opaque plate blocking begging apertures in front of the experimenter. NR.a: No Response, first 10s; NR.b: No Response, last 10s; WR.a: Wrong Response, first 10s; WR.b: Wrong Response, last 10s; C-: Experimenter absent. GLMM: Generalized Linear Mixed Model; LMM: Linear Mixed Model

Effect of recipient's presence and attentional state

Model 1

Number of begging gestures ~ Condition + (1 | Session) + (1 | Order) + (1 | Individual)
GLMM negative Binomial

Random effects

	Variance	Standard deviation
Individual	0.097	0.312
Order	0.000	0.000
Session	0.036	0.190

Fixed effects

	Estimate	Standard error	Z value	P value (with fdr correction)
C+ vs HU	-0.177	0.119	-1.487	0.196
C+ vs BT	-3.938	0.566	-6.954	< 0.001
C+ vs BTP	-3.651	0.493	-7.401	< 0.001
C+ vs C-	-18.147	4.635	-3.915	< 0.001
HU vs BT	-3.761	0.568	-6.624	< 0.001
HU vs BTP	-3.474	0.517	-6.720	< 0.001
HU vs C-	-22.115	452.855	-0.049	0.984
BT vs BTP	0.288	0.690	0.417	0.846
BT vs C-	-14.202	4.958	-2.865	0.007
BTP vs C-	-14.489	701.152	-0.021	0.984

Model 2

Latency for first begging gesture ~ Condition + (1 | Session) + (1 | Order) + (1 | Individual)
GLMM Gamma

Random effects

	Variance	Standard deviation
Individual	0.000	0.000
Order	0.000	0.000
Session	0.000	0.000

Fixed effects

	Estimate	Standard error	Z value	P value (with fdr correction)
C+ vs HU	-0.103	0.418	-0.247	0.960
C+ vs BT	-1.518	0.306	-4.964	< 0.001
C+ vs BTP	-1.520	0.306	-4.969	< 0.001
HU vs BT	-1.415	0.287	-4.940	< 0.001
HU vs BTP	-1.416	0.286	-4.945	< 0.001
BT vs BTP	-0.001	0.028	-0.050	0.960

Model 3

Number of upward gazes ~ Condition + (1 | Session) + (1 | Order) + (1 | Individual)

GLMM Poisson

Random effects

	Variance	Standard deviation
Individual	0.000	0.000
Order	0.000	0.000
Session	0.000	0.000

Fixed effects

	Estimate	Standard error	Z value	P value (with fdr correction)
C+ vs HU	1.758	0.484	3.630	< 0.001
C+ vs C-	0.216	0.606	0.357	0.721
HU vs C-	-1.542	0.449	-3.437	< 0.001

Model 4

Time spent away from begging sides ~ Condition + (1 | Session) + (1 | Order) + (1 | Individual)

LMM

Random effects

	Variance	Standard deviation
Individual	0.645	0.803
Order	0.066	0.257
Session	0.000	0.000

Fixed effects

	Estimate	Standard error	df	t value	P value (with fdr correction)
C+ vs HU	0.284	0.496	120.558	0.573	0.568
C+ vs BT	2.578	0.493	128.954	5.231	< 0.001
C+ vs BTP	2.662	0.490	129.346	5.434	< 0.001
C+ vs C-	4.025	0.567	5.864	7.095	< 0.001
BT vs HU	-2.294	0.494	127.384	-4.645	< 0.001
BT vs BTP	0.084	0.491	129.979	0.172	0.864
BT vs C-	1.447	0.567	5.949	2.554	0.044
BTP vs HU	-2.378	0.493	128.356	-4.821	< 0.001
BTP vs C-	1.363	0.566	6.013	2.409	0.053
HU vs C-	3.741	0.568	5.834	6.587	< 0.001

Effect of no response from the recipient

Model 5

Number of begging gestures ~ Condition + (1 | Session) + (1 | Order) + (1 | Individual)

GLMM Poisson

Random effects

	Variance	Standard deviation
Individual	0.135	0.367
Order	0.000	0.000
Session	0.042	0.206

Fixed effects

	Estimate	Standard error	Z value	P value
NR.a vs NR.b	-0.709	0.153	-4.624	< 0.001

Model 6

Proportion of lessened gestures ~ Condition + (1 | Session) + (1 | Order) + (1 | Individual)

LMM

Random effects

	Variance	Standard deviation
Individual	0.000	0.000
Order	0.000	0.000
Session	0.000	0.000

Fixed effects

	Estimate	Standard error	df	t value	P value
NR.a vs NR.b	0.063	0.058	48	1.082	0.285

Model 7

Proportion of amplified gestures ~ Condition + (1 | Session) + (1 | Order) + (1 | Individual)

LMM

Random effects

	Variance	Standard deviation
Individual	0.017	0.131
Order	0.000	0.000
Session	0.000	0.000

Fixed effects

	Estimate	Standard error	df	t value	P value
NR.a vs NR.b	0.106	0.066	36.791	1.619	0.114

Model 8

Proportion of audible begging gestures ~ Condition + (1 | Session) + (1 | Order) + (1 | Individual)

LMM

Random effects

	Variance	Standard deviation
Individual	0.045	0.211
Order	0.001	0.038
Session	0.000	0.000

Fixed effects

	Estimate	Standard error	df	t value	P value
NR.a vs NR.b	0.011	0.075	40.8992	0.153	0.880

Model 9

Change of begging aperture when gesturing ~ Condition + (1 | Session) + (1 | Order) + (1 | Individual)

GLMM Binomial

Random effects

	Variance	Standard deviation
Individual	0.032	0.180
Order	0.000	0.000
Session	0.000	0.000

Fixed effects

	Estimate	Standard error	Z value	P value
NR.a vs NR.b	-0.449	0.776	-578	0.563

Model 10

Number of gaze alternations ~ Condition + (1 | Session) + (1 | Order) + (1 | Individual)

GLMM negative Binomial

Random effects

	Variance	Standard deviation
Individual	0.096	0.309
Order	0.000	0.000
Session	0.078	0.279

Fixed effects

	Estimate	Standard error	Z value	P value
NR.a vs NR.b	-0.621	0.248	-2.502	0.012

Effect of a wrong response from the recipient

Model 11

Number of begging gestures ~ Condition + (1 | Session) + (1 | Order) + (1 | Individual)

GLMM Poisson

Random effects

	Variance	Standard deviation
Individual	0.115	0.339
Order	0.012	0.108
Session	0.000	0.000

Fixed effects

	Estimate	Standard error	Z value	P value (with fdr correction)
C+ vs WR.a	-2.265	0.264	-8.577	< 0.001
C+ vs WR.b	-2.093	0.245	-8.543	< 0.001
WR.a vs WR.b	0.172	0.337	0.511	0.610

Model 12

Latency for first begging gesture ~ Condition + (1 | Session) + (1 | Order) + (1 | Individual)

GLMM Gamma

Random effects

	Variance	Standard deviation
Individual	0.000	0.000
Order	0.000	0.000
Session	0.000	0.000

Fixed effects

	Estimate	Standard error	Z value	P value (with fdr correction)
C+ vs WR.a	-1.496	0.279	-5.356	< 0.001
C+ vs WR.b	-1.497	0.279	-5.358	< 0.001
WR.a vs WR.b	0.000	0.031	-0.016	0.987

Model 13
Proportion of lessened gestures ~ Condition + (1 | Session) + (1 | Order) + (1 | Individual)
LMM

Random effects

	Variance	Standard deviation
Individual	0.029	0.170
Order	0.017	0.129
Session	0.000	0.000

Fixed effects

	Estimate	Standard error	df	t value	P value (with fdr correction)
C+ vs WR.a	0.333	0.112	44.189	2.978	0.014
C+ vs WR.b	0.266	0.100	43.172	2.669	0.016
WR.a vs WR.b	-0.068	0.114	34.084	-0.595	0.556

Model 14
Number of gaze alternations ~ Condition + (1 | Session) + (1 | Order) + (1 | Individual)
GLMM Poisson

Random effects

	Variance	Standard deviation
Individual	0.373	0.611
Order	0.041	0.202
Session	0.049	0.222

Fixed effects

	Estimate	Standard error	Z value	P value (with fdr correction)
C+ vs WR.a	-1.226	0.235	-5.220	< 0.001
C+ vs WR.b	-0.970	0.215	-4.515	< 0.001
WR.a vs WR.b	0.256	0.266	0.962	0.336

For all experimental conditions

Model 15
Time spent in different locations ~ Location + (1| Session) + (1| Individual)
LMM

Random effects

	Variance	Standard deviation
Individual	0.000	0.000
Session	0.000	0.000

Fixed effects

	Estimate	Standard error	df	t value	P value
Front vs Back	5.867	0.431	238.000	13.607	< 0.001
